# Drug-Loadable Calcium Alginate Hydrogel System for Use in Oral Bone Tissue Repair

**DOI:** 10.3390/ijms18050989

**Published:** 2017-05-06

**Authors:** Luyuan Chen, Renze Shen, Satoshi Komasa, Yanxiang Xue, Bingyu Jin, Yepo Hou, Joji Okazaki, Jie Gao

**Affiliations:** 1 College of Stomatology, Southern Medical University, 1838 Guangzhou Avenue North, Guangzhou 510515, China; chen-luyuan900115@foxmail.com (L.C.); xue-yanxiang123@foxmail.com (Y.X.); bingyu_jin@outlook.com (B.J.); houyepo9626@foxmail.com (Y.H.); 2 Department of Removable Prosthodontics and Occlusion, Osaka Dental University, 8-1, Kuzuhahanazonocho, Hirakata, Osaka 573-1121, Japan; komasa-s@cc.osaka-dent.ac.jp (S.K.); joji@cc.osaka-dent.ac.jp (J.O.); 3 Department of Stomatology, Zhongshan Hospital, Medical College of Xiamen University, Xiamen 361000, China; shenrenze123@foxmail.com; 4 Department of Endodontics, Guangzhou Medical University, 195 Dongfeng West Road, Guangzhou 510000, China

**Keywords:** tissue engineering, drug-loadable system, calcium alginate hydrogels, human periodontal ligament cells, biocompatibility

## Abstract

This study developed a drug-loadable hydrogel system with high plasticity and favorable biological properties to enhance oral bone tissue regeneration. Hydrogels of different calcium alginate concentrations were prepared. Their swelling ratio, degradation time, and bovine serum albumin (BSA) release rate were measured. Human periodontal ligament cells (hPDLCs) and bone marrow stromal cells (BMSCs) were cultured with both calcium alginate hydrogels and polylactic acid (PLA), and then we examined the proliferation of cells. Inflammatory-related factor gene expressions of hPDLCs and osteogenesis-related gene expressions of BMSCs were observed. Materials were implanted into the subcutaneous tissue of rabbits to determine the biosecurity properties of the materials. The materials were also implanted in mandibular bone defects and then scanned using micro-CT. The calcium alginate hydrogels caused less inflammation than the PLA. The number of mineralized nodules and the expression of osteoblast-related genes were significantly higher in the hydrogel group compared with the control group. When the materials were implanted in subcutaneous tissue, materials showed favorable biocompatibility. The calcium alginate hydrogels had superior osteoinductive bone ability to the PLA. The drug-loadable calcium alginate hydrogel system is a potential bone defect reparation material for clinical dental application.

## 1. Introduction

Periodontal diseases are among the most common chronic disorders that have plagued humans for centuries [1]. One of the principal pathological changes associated with periodontitis is alveolar resorption [2,3]. The resorption of alveolar bone leads to a loss of periodontal supporting tissue. If periodontal disease is not treated properly, it eventually causes the gradual loosening of teeth. Lack of alveolar bone tissue may also cause great difficulties during the design and operation of denture restoration and implant surgery [4].

At present, the clinical treatment for periodontitis mainly involves ultrasonic, supragingival, and subgingival scaling. However, periodontal bone tissue regeneration still relies upon the application of tissue regeneration scaffold material. Scholars have used autologous bones, allograft bones, heterogeneous bones, and other bone regeneration materials to repair periodontal bone tissue defects [5,6]. However, during the process of guided bone regeneration, traditional bone scaffolds may cause problems such as bone donor site trauma, the larger wounding of the implant area, and aseptic inflammation. Furthermore, oral bone tissue reparation has unique characteristics; when biomaterials are implanted to repair bone defects, we should simultaneously consider the effect of periodontal soft tissue and hard tissue and not only that of the direct implantation of the bone defect reparation material. Additionally, the oral environment is home to bacteria and it is easy to cause bacteria adhesion and wound infection. Therefore, identifying an artificial material that can load drugs with favorable biological properties is crucial.

Since Rokkanen [7] first employed the absorbable material PGA (polyglycolic acid) clinically in 1985, it has been the subject of considerable clinical research and application; however, traditional biodegradable absorbent materials are still limited by their biomechanics, biocompatibility, and other factors. The traditional tissue-engineering materials used in treatment such as polylactic acid (PLA) and poly-l-lactide, which, it has been reported, could be printed by a 3D printer [8,9], have acidic degradation products and may lead to aseptic inflammation. Currently, drug-loadable systems are being widely investigated in the field of degradable biomaterials. Such systems can store drugs or proteins and then release them in the defective region as biodegradable materials. Hence, drug-loadable systems can be combined with different drugs according to the unique characteristics of the defective area to decrease healing time [10,11,12].

Hydrogels, which are currently used in drug-loadable systems, are increasingly employed in regenerative medicine due to their favorable biocompatibility and potential for injection into defect tissue in a minimally invasive manner. Injectable hydrogels containing drugs can also simply be injected under the skin or into a target area, immediately forming a depot for clinical treatment [13,14]. Hydrogels usually comprise polymers such as chitosan and alginate [15,16]. Alginate is an attractive, naturally occurring anionic and hydrophilic polysaccharide that contains blocks of (1-4)-linked β-d-mannuronic acid (M) and α-l-guluronic acid (G) monomer [17]. Previous studies have reported numerous methods for preparing hydrogels, including ionic crosslinking [18], phase transition [19], cell crosslinking [20], free radical polymerization [21], and “click” reactions [22]. Ionic crosslinking is one of the most commonly used methods for preparing alginate hydrogels. Alginate is easily soluble in water or culture medium and is gelated using divalent cations such as Ca^2+^, Mg^2+^, or Fe^2+^. Among these cations, Ca^2+^ is the most widely used for the ionic crosslinking of alginate [23]. Calcium chloride (CaCl_2_) has been demonstrated to be one of the best Ca^2+^ sources during this procedure.

Calcium alginate hydrogels have a three-dimensional (3D) network structure that can carry drugs and proteins that have tissue regeneration benefits, and can then release these drugs or proteins stably at defect sites [24,25]. Previous studies have reported that calcium alginate hydrogel can stimulate the proliferation, differentiation, and maturation of cultured osteoblasts in vitro [26,27].

Drug-loadable calcium alginate hydrogel may thus be suitable for oral tissue regeneration involving complex environments. However, few studies have investigated the effect of using calcium alginate hydrogels during oral tissue reparation. The regeneration of periodontal tissue includes both bone tissue healing and periodontal healing. Both human periodontal ligament cells (hPDLCs) and bone marrow stromal cells (BMSCs) play crucial roles in the regeneration process. Previous studies have reported that hPDLCs have regenerative potential for multilinear differentiation and can be used as seed cells for periodontal regeneration [28,29]. BMSCs, which promote bone tissue regeneration, can be employed as seed cells for bone tissue reparation. Therefore, we selected both hPDLCs and BMSCs as the research objects in this study because of the unique characteristics of oral tissue.

In this study, a drug-loadable calcium alginate hydrogel system for use in oral tissue reparation that can carry suitable drug concentrations was developed. The swelling ratio, degradation time, and BSA release rate of the material were measured. Finally, we evaluated the biocompatibility of the drug-loadable calcium alginate hydrogel using in vivo and in vitro experiments.

## 2. Results

### 2.1. Material Preparation

**Figure 1:**
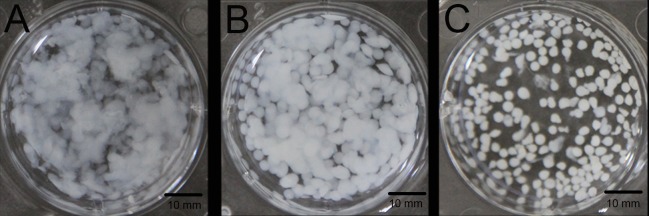
Gross appearance of hydrogels with calcium alginate concentration (**A**) 12.5 mg/mL; (**B**) 25 mg/mL; and (**C**) 50 mg/mL.

**Figure 2:**
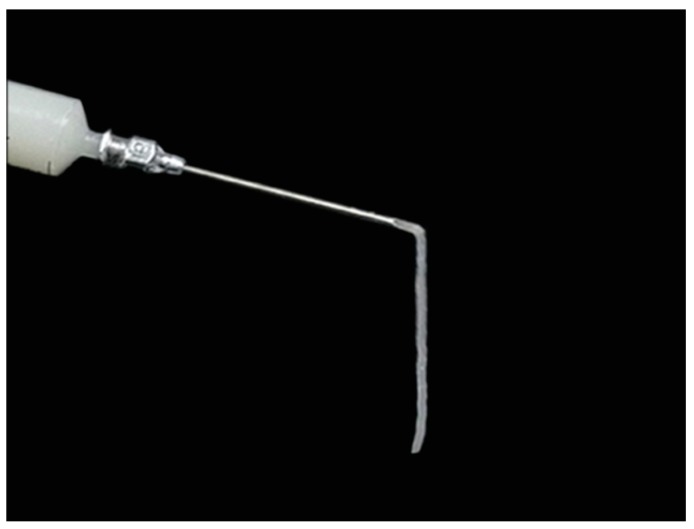
Injectable calcium alginate hydrogel (concentration 25 mg/mL).

Figure 1 presents gross appearance images of the 12.5, 25, and 50 mg/mL calcium alginate hydrogels. The hydrogel containing 12.5 mg/mL calcium alginate had a white floc appearance; however, the hydrogel with a concentration of 50 mg/mL had a grainy appearance. Different concentrations thus resulted in hydrogels with different properties. The outflow volume was lowest in the 50 mg/mL calcium alginate hydrogel and highest in 12.5 mg/mL calcium alginate hydrogel. Additionally, the formability of the hydrogels increased as the concentration was increased. Injectable drug-loaded hydrogels were successfully prepared with a calcium alginate concentration of 25 mg/mL (Figure 2).

### 2.2. Swelling Ratio, Degradation, and BSA Release Assay

**Figure 3:**
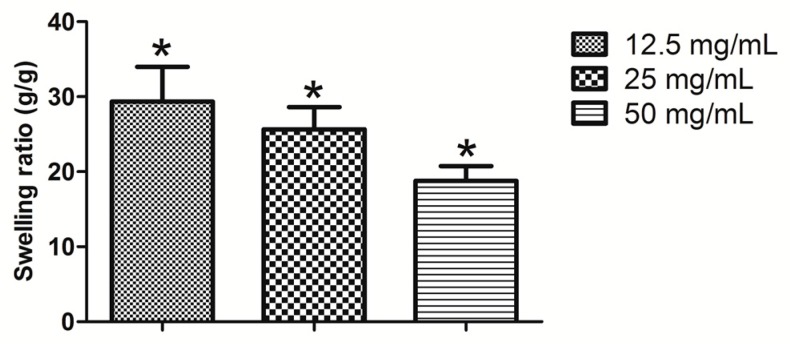
Swelling ratio of calcium alginate hydrogels (* *p* < 0.05 *n* = 5).

**Figure 4:**
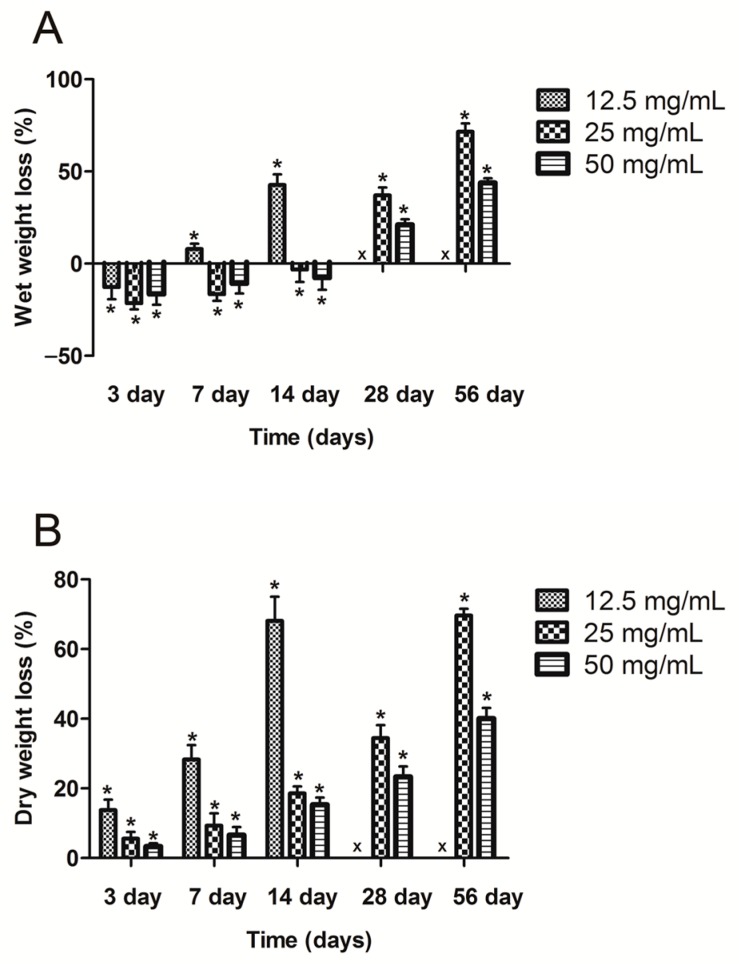
(**A**) Wet and (**B**) dry weight loss rates (* *p* < 0.05 *n* = 5). “x” above 28 and 56 days means samples had finished degradation.

**Figure 5:**
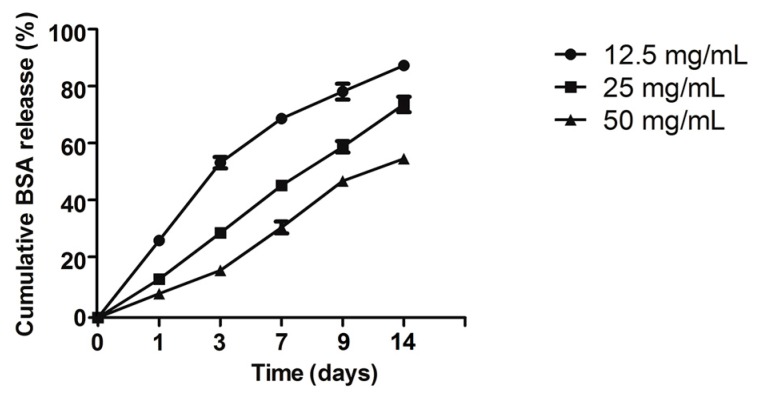
Cumulative BSA release of calcium alginate hydrogels (*n* = 5).

The swelling ratio of different concentration calcium alginate hydrogels is illustrated in Figure 3. The swelling ratio of the 12.5 mg/mL hydrogel was significantly higher than that of the 50 and 25 mg/mL hydrogels (*p* < 0.05); however, the 12.5 mg/mL hydrogel could not be molded easily after it had absorbed water. During the first three days, the wet weight of all the calcium alginate hydrogels increased, resulting in wet weight loss rates of less than zero; after three days, the wet weight loss rate gradually increased (Figure 4A). After four weeks of degradation in PBS, the hydrogel containing 12.5 mg/mL calcium alginate had almost finished degradation, and its wet and dry weights could therefore not be measured. Unlike their wet weight loss, the dry weight loss increased continuously (Figure 4B). The results of the BSA release test revealed that the 12.5 mg/mL hydrogel released more initial BSA than the other hydrogels, but that its sustained release ability was inferior to those of the 25 and 50 mg/mL hydrogels; however, the initial release ability of the 50 mg/mL hydrogel was poor (Figure 5).

### 2.3. Culture and Proliferation Assay of hPDLCs

**Figure 6:**
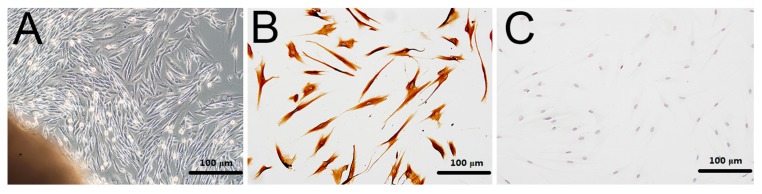
Initiation culture and immunohistochemical identification of hPDLCs (200×): (**A**) hPDLCs migrated from the border of the tissue; (**B**) anti-vimentin positive in hPDLCs; (**C**) anti-keratin negative in hPDLCs.

From Figure 6A, the hPDLCs that had migrated from the tissue were observed. The fourth to sixth passages of hPDLCs were identified using immunohistochemical staining (Figure 6B,C).

**Figure 7:**
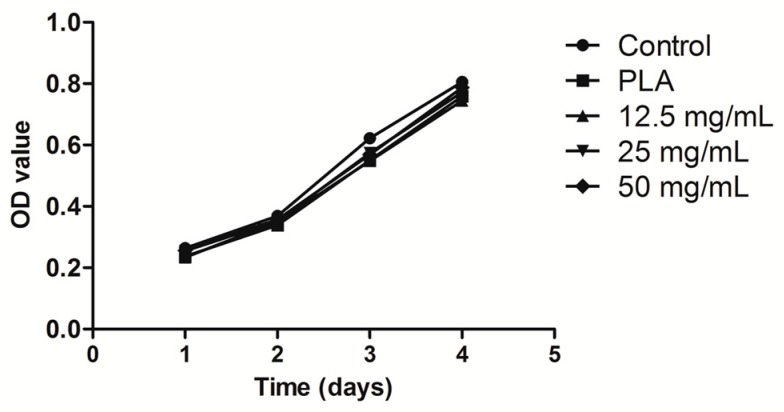
Growth curve of hPDLCs.

**Figure 8:**
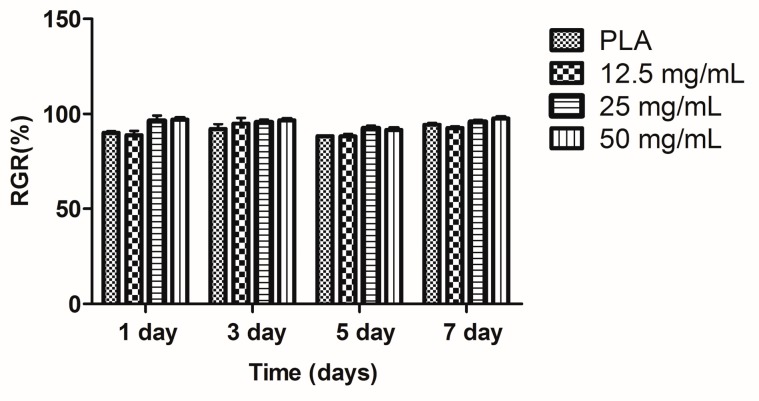
RGR (%) of co-cultured hPDLCs.

The hPDLCs were cultured jointly with calcium alginate hydrogels. The MTT results demonstrated that the calcium alginate concentration of a hydrogel may affect the proliferation of hPDLCs (Figure 7), but the RGR of all the samples was higher than 80% (Figure 8). According to the cytotoxicity grading criteria ISO 10993-5:2009 (E) presented in Table 1 [30], the cytotoxicity grade of all the calcium alginate hydrogels was grade 1 (Table 2), indicating that these materials had favorable biocompatibility.

### 2.4. Alizarin Red Staining

**Figure 9:**
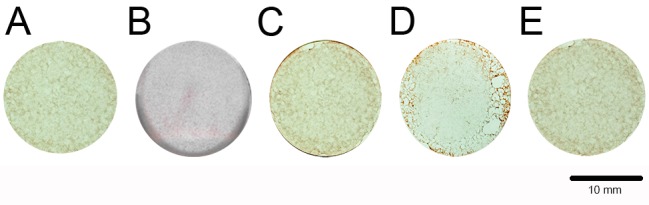
Mineralization nodules of BMSCs: (**A**) Control; (**B**) PLA; (**C**) 12.5 mg/mL; (**D**) 25 mg/mL; (**E**) 50 mg/mL.

**Figure 10:**
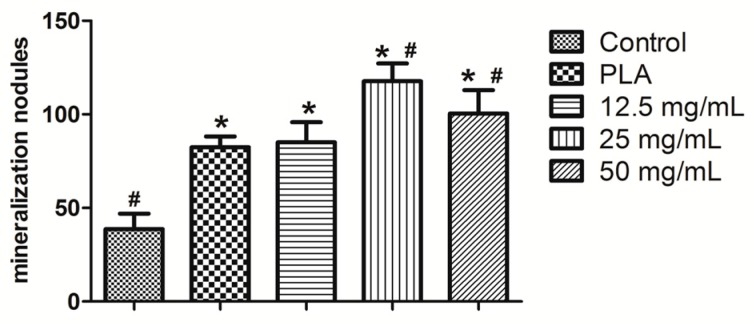
Number of mineralization nodules (* *p* < 0.05 vs. control; # *p* < 0.05 vs. PLA).

Figure 9 presents the results of alizarin red staining. Mineralization capacity was measured by counting the mineralized nodules (Figure 10). Both PLA and the calcium alginate hydrogels were discovered to have more mineralized nodules than the control group, whereas the 25- and 50-mg/mL hydrogels had superior mineralization capacity to the PLA (*p* < 0.05). The hydrogel containing 25 mg/mL calcium alginate had a higher ability to induce BMSC mineralization than any other material.

### 2.5. Real-Time Quantitative PCR

**Figure 11:**
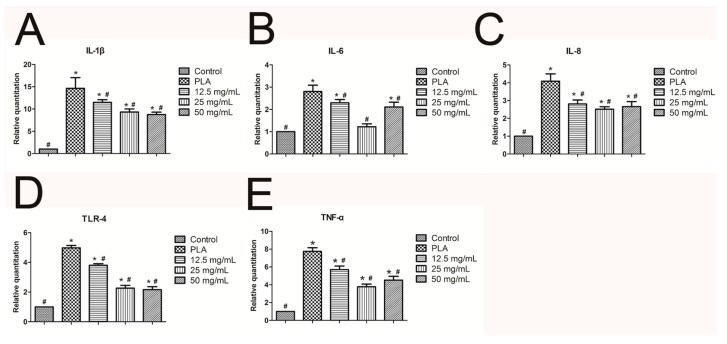
Expression of inflammation-related genes of hPDLCs (* *p* < 0.05 vs. control, # *p* < 0.05 vs. PLA): (**A**) *IL-1*β; (**B**) *IL-6*; (**C**) *IL-8*; (**D**) *TLR-4*; (**E**) *TNF-*α.

**Figure 12:**
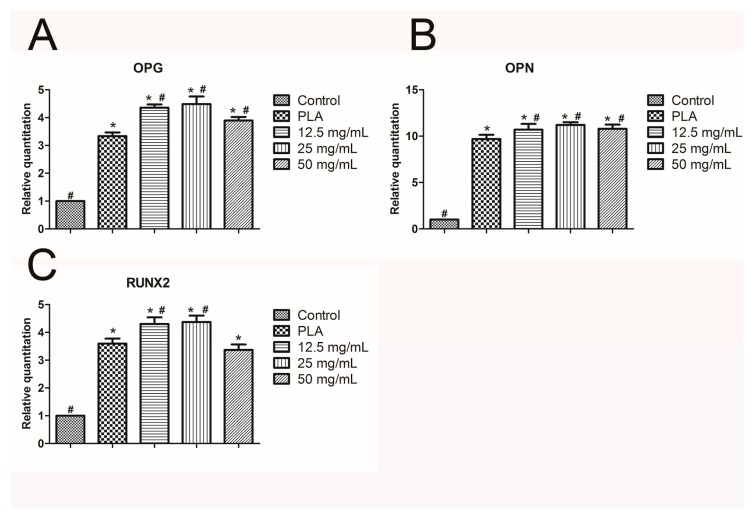
Expression of osteogenesis-related genes (* *p* < 0.05 vs. control, # *p* < 0.05 vs. PLA): (**A**) *OPG*; (**B**) *OPN*; (**C**) *RUNX2*.

The calcium alginate hydrogel and PLA scaffold promoted the expression of the mRNA of hPDLCs in IL-1β, IL-6, IL-8, TLR4, and TNF-α (Figure 11A–E, respectively). The inflammatory reaction induced by the calcium alginate hydrogels was significantly smaller than that induced by control group and the PLA (*p* < 0.05) Compared with the control group, the calcium alginate hydrogel and PLA both promoted the mRNA expression of OPG, OPN, and RUNX2 (*p* < 0.05) in bone marrow mesenchymal stem cells, and the effect was strongest when the calcium alginate concentration was 25 mg/mL (*p* < 0.05; Figure 12A–C).

### 2.6. Histological Observation

**Figure 13:**
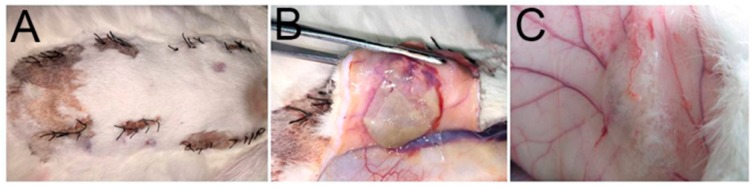
Gross appearances of samples: (**A**) wound; (**B**) PLA; (**C**) hydrogels.

**Figure 14:**
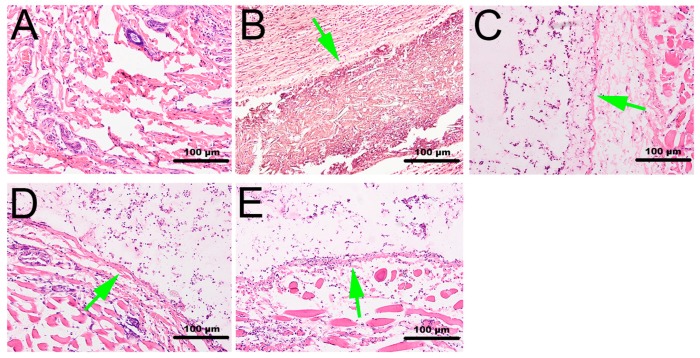
HE staining results (green arrows show the dividing line between materials and tissue): (**A**) control; (**B**) PLA; (**C**) 12.5 mg/mL hydrogel; (**D**) 25 mg/mL hydrogel; (**E**) 50 mg/mL hydrogel.

To evaluate the initial biocompatibility of the calcium alginate hydrogels in vivo, the materials were implanted into rabbits for seven days, after which tissues were surgically removed and stained using the HE method and the inflammation reactions observed. The gross appearance of the samples (Figure 13) revealed an evident tuber at the implant site; the wound was found to have healed well, and neither necrosis of tissue, hyperemia, nor hemorrhaging were observed in the calcium alginate hydrogel groups. In the PLA group, however, we discovered congestion. Furthermore, the HE results (Figure 14) demonstrated that the number of inflammatory cells in the control group, PLA scaffold group, and calcium alginate hydrogel groups had increased, but also that there were gaps between the PLA scaffolds and the tissue. The calcium alginate hydrogel–tissue boundaries were all clear.

### 2.7. Mandibular Defect Model and Micro-CT Scanning

**Figure 15:**
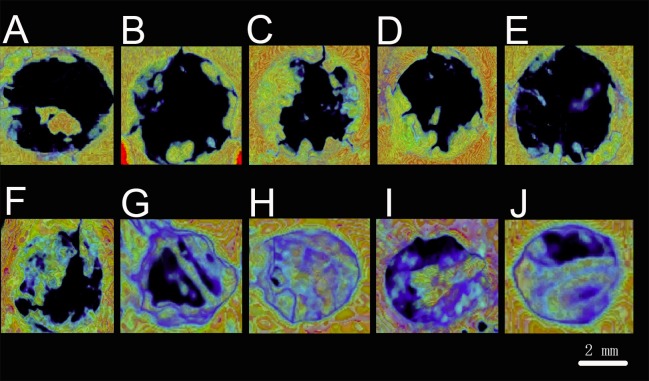
Transverse reconstructed micro-CT images: after seven days (**A**–**E**) and 28 days (**F**–**J**); (**A**,**F**) control; (**B**,**G**) PLA; (**C**,**H**) 12.5 mg/mL hydrogel; (**D**,**I**) 25 mg/mL hydrogel; (**E**,**J**) 50 mg/mL hydrogel.

**Figure 16:**
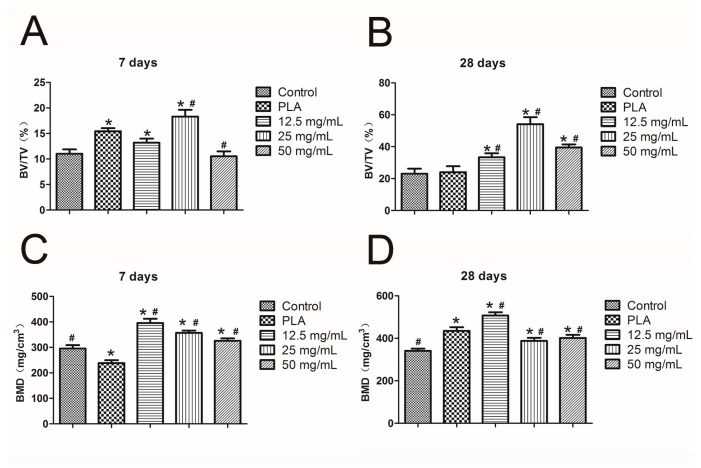
Micro-CT quantitative evaluation within the ROI (* *p* < 0.05 vs. control, # *p* < 0.05 vs. PLA): (**A**) 7 days and (**B**) 28 days BV/TV; (**C**) 7 days and (**D**) 28 days BMD.

Reconstructed 3D micro-CT images of the mandibles are presented in Figure 15. The cortical is marked green, whereas the cancellous bone is marked blue. The BV/TV data (Figure 16A,B) were used to quantitatively assess the newly formed bone in the mandibular defect sites. After seven days of surgical implantation, the PLA, 12.5- and 25-mg/mL groups had significantly higher BV/TV than the control group (*p* < 0.05). Less bone was formed in the PLA and control groups after 28 days of implantation compared with the hydrogel groups (*p* < 0.05). The most bone was discovered to form in the 25-mg/mL group (*p* < 0.05).

## 3. Discussion

A drug-loaded regenerative material should have two main characteristics: It should be able to carry proteins or other osteogenic factors and release them in a sustained manner at the defect site, and it should have essential characteristics such as excellent biocompatibility, plasticity, degradability, and osteogenic activity. Our study successfully prepared drug-loaded injectable hydrogels containing a suitable concentration of calcium alginate and evaluated their biocompatibility for periodontal bone tissue reparation through in vitro and in vivo experiments. Drug-loaded calcium alginate hydrogels were demonstrated to be suitable regenerative biomaterials for the reparation of oral bone defects, and the appropriate concentration of calcium alginate in the hydrogel, for use in an injectable system, was identified as 25 mg/mL.

In this study, we successfully prepared calcium alginate hydrogels and discovered that the hydrogels’ liquidity decreased and formability increased as their calcium alginate concentration increased. These results may have been due to increased cross-linking interactions between Ca^2+^ and the sodium alginate in the high-concentration hydrogels, which changed the hydrogels’ stability.

The calcium alginate hydrogels could store water or drugs and could mimic the features of the extracellular matrix due to their 3D-pore-network structure, which was formed by cross-link reactions [31,32]. The swelling ratio, BSA release assay, and degradation results are explained by the formation of a 3D-network structure in the calcium alginate hydrogels. Such a structure can store water, proteins, or drugs in its pores. However, the pore size of the 3D network decreased as the calcium alginate concentration was increased [33]. Larger pores can store more water or drugs, but also result in decreased stability. As a consequence, the 12.5 mg/mL hydrogel degraded much faster than the 25 or 50 mg/mL hydrogel. During the bone healing process, a hard callus formed as osteoblast cells created new bone, adding minerals to harden it. This stage typically begins two weeks after implantation. The degradation time of the 25 mg/mL group was equal to the bone healing time, which indicated that the 25 mg/mL calcium alginate hydrogel could make a higher contribution to the oral bone tissue regeneration than the 12.5- or 50-mg/mL hydrogels. Drugs are stored in drug-loaded systems in different ways. BSA can be stored directly in the hydrogels’ 3D pore network structure. However, the positively charged functional group in BSA can combine with the carboxyl groups in alginate and ultimately form BSA/alginate complexes, and the release of BSA from such hydrogels is sustained as the degradation of the hydrogel proceeds [34]. Calcium alginate hydrogels can thus combine with drugs or proteins in a variety of ways, making them ideal for drug delivery.

The hPDLC proliferation result can be explained by an excessively high concentration of Ca^2+^ produced during degradation or by calcium chloride residue in the hydrogels. One study reported that an excessively high concentration of Ca^2+^ may lead to the inhibition of cell proliferation [35]. The alginate hydrogels degraded enzymatically through a process involving the loss of divalent ions such as Ca^2+^ into the surrounding medium [36]. The 12.5-mg/mL group hydrogels degraded quickly, which resulted in an excessively high concentration of Ca^2+^ in the medium, ultimately causing proliferation inhibition. However, the biosafety of the three calcium alginate hydrogels were all graded 1, which indicated that the calcium alginate hydrogels were sufficiently biocompatible for use in the periodontium.

Toll-like receptors (TLRs) play an essential role in inflammatory injury and aseptic inflammation [37]. TLR4 has been reported as a critical TLR in initial inflammation reactions. TLR4 combines with a protein adapter named myeloid differentiation factor 88 (MyD88) and then activates the nuclear factor-κB (NF-κB), promoting the production of inflammation-related cytokines such as IL-6, IL-8, TNF-α, and IL-1β [38]. Our study discovered that both PLA and the calcium alginate hydrogels promoted the expression of the inflammation-related factors TLR4, IL-6, IL-8, TNF-α, and IL-1β, but the calcium alginate hydrogels resulted in less inflammation than the PLA. This result inferred that the aseptic inflammation caused by the calcium alginate hydrogels may be produced by the TLR4/MyD88/NF-κB pathway. Therefore, in future studies we can combine drugs that inhibit the TLR4/MyD88/NF-κB pathway to reduce aseptic inflammation.

The calcium alginate hydrogels caused less hematoma than the PLA. Due to their high swelling ratio, the calcium alginate hydrogels could absorb wound exudation; however, an increase in calcium ion concentration is a major cause of platelet activation, which promotes blood clotting [39]. Ca^2+^ can also be mobilized by various secondary messengers, and these messengers act on the specific receptors and channels that contribute to blood clotting such as IP3 receptors, ryanodine receptors, and two-pore channels [40,41]. In this study, all incisions made were of the same size in order that the effect of the PLA and calcium alginate hydrogels could be accurately compared. A previous report indicated that injectable hydrogel systems may require less healing time than traditional scaffolds [42]. This can be explained by the major disadvantage of traditional tissue-repairing materials such as PLA or PGA scaffolds; these traditional scaffolds do not achieve a true 3D structure and cause minimal invasive trauma compared with injectable hydrogels. When calcium alginate hydrogels are prepared as the injectable system, less healing time may be required. The HE results indicated that the PLA scaffolds could not combine with the soft tissue as effectively as the calcium alginate hydrogels during the initial stage after implantation.

Calcium ions notably enhance the deposition of calcium salt and the formation of calcium nodes [43]. In this study, all of the three calcium alginate hydrogels improved the deposition of calcium nodules. Although the 12.5 mg/mL group had the highest concentration of Ca^2+^ ions, due to the over-inhibition of cell proliferation it had less mineralization nodules than the 25 mg/mL group. RUNX2 has been identified as an early osteogenic-related marker [44], and both OPN and OPG can promote osteogenic ability. Consistent with previous reports, our real-time PCR results for osteogenesis-related factors revealed that calcium alginate hydrogels promoted the expression of OPG, OPN, and RUNX2 [45,46,47]. Thus, the calcium alginate hydrogels accelerate mineralization in vitro. Our study also evaluated the osteogenesis-inducing ability of calcium alginate hydrogels in vivo. As in the in vitro experimental results, the in vivo experiments demonstrated that calcium alginate hydrogel could enhance osteogenesis and has superior osteoinductive bone ability than the traditional material (PLA). Due to its high release of Ca^2+^, the 12.5-mg/mL group had higher BMD than the other groups; however, the 25-mg/mL group exhibited the best osteogenesis ability. This may be explained by its suitable degradation time and calcium ion concentration.

A mandibular defect model was used to evaluate bone tissue regeneration. Although this model cannot fully simulate the complex situation in humans, it has been previously used to prove the osteogenesis ability of biomaterial [48,49]. Further detailed studies of calcium alginate hydrogels are needed to improve their clinical applications, such as in drug-loaded injectable systems or 3D printing systems with various drugs.

## 4. Materials and Methods

### 4.1. Material Preparation

Deionized water was used to prepare sodium alginate (Sigma-Aldrich, St. Louis, MO, USA) solutions with concentrations of 12.5, 25, and 50 mg/mL. Calcium chloride (0.6%; Sigma-Aldrich) was used as a cross-linking agent. Sodium alginate and calcium chloride were simultaneously injected into a helical pipe, and three concentrations of calcium alginate solution were prepared. Pure PLA was fully dissolved in chloroform solvent. An electrospinning unit (Kato Tech Co., Ltd., Kyoto, Japan) was used to perform emulsion electrospinning, and the feed rate of the emulsion spinning dope was 0.012 mL/min. A voltage of 17 kV was applied to the collector.

### 4.2. Swelling Ratio, Degradation, and BSA Release Assay

Equal weights of calcium alginate hydrogels were prepared for measuring their wet weight (*W*_w_) and dry weight (*D*_w_). The swelling ratio was defined as follows:

(1) $$\mathrm{Swelling}\;\mathrm{ratio}=\frac{W_{w}-D_{w}}{D_{w}}$$

Degradation was evaluated using wet weight loss (%) and dry weight loss (%). The samples were removed from phosphate-buffered saline (PBS) solution after 3, 7, 14, and 28 days of degradation, and their wet and dry weights were measured. The wet and dry weight losses of the calcium alginate hydrogels were calculated as follows:

(2) $$\mathrm{Wet}\;\mathrm{weight}\;\mathrm{loss}\left(\%\right)=\frac{W_{w0}-W_{\mathrm{wt}}}{W_{w0}}\times 100\%$$

(3) $$\mathrm{Dry}\;\mathrm{weitht}\;\mathrm{loss}\left(\%\right)=\frac{W_{d0}-W_{\mathrm{dt}}}{W_{d0}}\times 100\%$$

Here, *W*_w0_ and *W*_wt_ are the weights of wet materials before and after degradation, respectively. *W*_d0_ and *W*_dt_ are the weights of dry materials before and after degradation, respectively.

Sodium alginate and bull serum albumin (BSA) (50 μg/mL) were mixed, dissolved in deionized water, and then added to calcium chloride solution for ion exchange. The resultant calcium alginate gel/BSA samples were dispersed in a tube and 1 mL of PBS solution was added, after which the mixture was incubated at 37 °C in a shaking bath for 1, 2, 4, 6, 9, or 14 days. Bicinchoninic acid assay (BCA) was used to determine the BSA concentrations in the supernatants by testing the optical density (OD) value at 570 nm. The BSA release profiles were then used to draw the curve.

### 4.3. Culture and Proliferation Assay of hPDLCs

Periodontal tissue was collected from donors in our hospital. A well-documented method was used to culture hPDLCs [50]. Ethical approval for this investigation was obtained from the Human Research Ethics Committee, Southern Medical University, China (approval no. NFYY-2015-84). The fourth to sixth passages of hPDLCs were examined for the following experiment. Cell proliferation was determined using 12.5, 25, and 50 mg/mL calcium alginate hydrogels as the experimental groups, which were co-cultured with hPDLCs for 1, 3, 5, or 7 days in 96-well plates. Control groups and PLA groups were also employed. MTT assay was used to observe the proliferation of hPDLCs. MTT solution (20 μL) was added to 96-well plates and kept at 37 °C under a 5% CO_2_ flow for 4 h. The solution was then discarded and 150 μL of dimethyl sulfoxide was added to each well and the well plate was shocked for 10 min. Finally, the OD was detected using spectrophotometry with source light of wavelength 490 nm and the toxicity of calcium alginate hydrogels was evaluated. The relative growth rates of the cells were calculated using.

(4) $$\mathrm{Relative}\;\mathrm{growth}\;\mathrm{rate}\;\left(\mathrm{RGR}\right)=\left(\frac{\mathrm{OD}\;\mathrm{of}\;\mathrm{test}\;\mathrm{sample}}{\mathrm{OD}\;\mathrm{of}\;\mathrm{control}}\right)\times 100\%$$

### 4.4. Alizarin Red Staining

Different concentrations of calcium alginate hydrogels and PLA scaffolds were co-cultured with BMSCs in 24-well plates for 14 days. Alizarin red staining was then employed to detect calcium deposits. First, 4% polyoxmethylene was added to the wells for 10 min. Then, alizarin red was added to each well for 15 min and the wells were subsequently washed twice using deionized water. Under a high-power microscope, 10 randomly selected fields of each sample and the number of mineralized nodules was counted.

### 4.5. Real-Time Quantitative Polymerase Chain Reaction (PCR)

Real-time quantitative PCR was used to determine how different concentrations of calcium alginate hydrogels affected the inflammatory reaction of hPDLCs and the osteogenesis-related factors of BMSCs. Total RNA was extracted using TRIzol^®^ Reagent in each well according to the manufacturer’s protocol and employing reverse transcription. Real-time PCR was performed using the PrimeScript^®^ RT reagent (Takara, Shiga, Japan) and SYBR Premix Dimer Eraser (Takara, Shiga, Japan) kits. Table 3 lists the primer sequences used in this study.

### 4.6. Histological Observation

New Zealand rabbits (six males, weighing approximately 2.5 kg) were implanted with PLA scaffolds and injected subcutaneously with different concentrations of calcium alginate hydrogels. All the procedures of the in vivo experiment were performed according to the national animal care guidelines. Hematoxylin–eosin (HE) staining was used to determine the initial biosecurity performance of the calcium alginate in the tissue seven days after injection and the tissue was also observed using a microscope.

### 4.7. Mandibular Defect Model and Micro-CT Scanning

Bone defects of diameter × height = 5 mm × 2 mm were prepared in the mandibles of New Zealand rabbits (six males weighing 2.5–3.0 kg). Three different concentrations of calcium alginate hydrogels were implanted in the bone defects; a PLA scaffold group and control group were also used, with the rabbits in these groups not receiving the injection. The rabbits were anesthetized and killed either seven or 28 days after surgical implantation. The explanted mandibles were scanned using micro-CT (SMX-130CT) at a voltage of 50 kV and a current of 200 μA. Tri/3D-BON software was used to construct three-dimensional images of the mandibles. A cylindrical region of interest (ROI) 5 mm in diameter within the repair site was positioned for analysis. Bone volume divided by total volume (BV/TV ratio) and bone mineral density (BMD) were used to evaluate bone regeneration in this study.

### 4.8. Statistical Analysis

SPSS 19.0 was used to perform statistical analysis. All results are expressed as mean ± standard deviation. The data were analyzed using one-way analysis of variance followed by the Student’s *t*-test for comparisons between groups. Significance was indicated by *p* < 0.05 in this study.

## 5. Conclusions

In this study, a drug-loaded hydrogel material for use in oral bone tissue regeneration was produced using ion crosslinking. Calcium alginate hydrogels have favorable water absorption capacity and BSA release ability. The 25-mg/mL hydrogel had a degradation time that was suitable for bone regeneration. The prepared calcium alginate hydrogel meets the biocompatibility requirements for use in oral bone tissue regeneration. Although the hydrogel promotes the expression of inflammatory cytokines, it causes less inflammatory reactions than traditional PLA scaffolds. Calcium alginate hydrogels promote the mineralization of BMSCs and the expression of osteogenesis-related cytokines. Our in vivo study results correlated with the in vitro study results. The inflammatory response of calcium alginate hydrogel in soft tissue was lower than that of PLA, but the osteoinductive bone ability of the calcium alginate hydrogel was better than that of the PLA. Therefore, the drug-loaded calcium alginate hydrogel is a potential bone defect reparation material for clinical dental application.

## Figures and Tables

**Table 1:** Cytotoxicity grade standards.

RGR (%)	Cytotoxicity Grade
100+	0 (non-poisonous, qualification)
75–99	1 (slightly poisonous, qualification)
50–74	2 (moderately poisonous, disqualification)
25–49	3 (severely poisonous, disqualification)
1–24	4 (disqualification)
0	5 (disqualification)

**Table 2:** Cytotoxicity grade of polylactic acid (PLA) and calcium alginate hydrogels.

RGR (%)	Cytotoxicity Grade
PLA	1
12.5 mg/mL	1
25 mg/mL	1
50 mg/mL	1

**Table 3:** Quantitative real-time PCR primer sequences.

Primer	Primer Sequence
β-actin	Forward primer: GCGCGGCTACAGCTTCA Reverse primer: TCTCCTTAATGTCACGCACGAT
IL-1β	Forward primer: ATAAGCCCACTCTACAGCT Reverse primer: ATTGGCCCTGAAAGGAGAGA
IL-8	Forward primer: GCTTTCTGATGGAAGAGAGC Reverse primer: GGCACAGTGGAACAAGGACT
IL-6	Forward primer: GTACCCCCAGGAGAAGATTC Reverse primer: CAAACTGCATAGCCACTTTC
TLR-4	Forward primer: TGAGGACCGACACACCAATG Reverse primer: TGCAATGGATCAAGGACCAG
TNF-α	Forward primer: TCTCATCAGTTCTATGGCCC Reverse primer: GGGAGTAGACAAGGTACAAC
OPG	Forward primer: CCCTTGCCCTGACCACTACTA Reverse primer: GCTTGCACCACTCCAAATCC
RUNX2	Forward primer: TGCTGGAGTGATGTGGTTTTCT Reverse primer: CCCCTGTTGTGTTGTTTGGTAA
OPN	Forward primer: TGAAACGAGTCAGCTGGATG Reverse primer: TGAAATTCATGGCTGTGGAA

## Abbreviations

BSA	Bovine serum albumin
MTT	Methyl Thagolyl Tetragoliam
PLA	Polylactic Acid
hPDLCs	Human Periodontal Ligament Cells
BMSCs	Bone Marrow Stromal Cells
PGA	Polyglycolic Acid
BCA	Bicinchoninic Acid Assay
PBS	Phosphate-buffered Saline
IL-1β	Interleukin-1 β
IL-6	Interleukin-6
IL-8	Interleukin-8
TNF-α	Tumor Necrosis Factor- α
NF-κB	Nuclear Factor-κ-gene Binding
MyD88	Myeloid Differentiation Factor-88
TLRs	Toll-like Receptors
TLR4	Toll-like Receptor-4
OPG	Osteoprotegerin
OPN	Osteopontin
RUNX2	Runt-related Transcription Factor 2
BV/TV	Bone Volume/Total Volume
BMD	Bone Mineral Density
PCR	Polymerase Chain Reaction
RGR	Relative growth rate
